# Glycosylation of the murine cardiac channel TRPM4 is altered by the pathogenic p.I376T variant

**DOI:** 10.1113/EP093873

**Published:** 2026-04-29

**Authors:** Sabrina Guichard, Emanuele di Lorenzo, Dominic Schneiter, Maria Essers, Prakash Arullampalam, Jean‐Sébastien Rougier, Hugues Abriel

**Affiliations:** ^1^ Institute of Biochemistry and Molecular Medicine University of Bern Bern Switzerland

**Keywords:** cardiac electrophysiology, murine knock‐in model, TRPM4 channel

## Abstract

TRPM4 is a calcium‐activated, voltage‐modulated, non‐selective cation channel expressed in various tissues, including the heart. In 2016, we reported on a large French family with progressive heart block type I carrying the variant TRPM4 p.I376T. In the present study, the aim was to investigate the consequence of the channel variant TRPM4 p.I376T in cardiac physiology in a newly generated *Trpm4* knock‐in mouse line. Male and female *Trpm4* knock‐in (*Trpm4* ^I376T/I376T^) and wild‐type mice of different young ages (12, 18, 24 and 36 weeks old) were phenotyped using surface ECGs. Western blots were performed to quantify TRPM4 protein surface expression in cardiac tissue. Finally, patch‐clamp experiments were conducted to quantify the ‘TRPM4 current’ from freshly isolated ventricular cardiomyocytes. Assessment of cardiac electrophysiology using surface ECGs indicated no significant differences between the two genotypes at any age. Western blot analyses revealed a significant decrease in the highly glycosylated fraction of the TRPM4 protein in *Trpm4* ^I376T/I376T^ hearts compared with wild‐type tissues. However, this alteration did not influence the ‘TRPM4 current’ when comparing *Trpm4* ^I376T/I376T^ and wild‐type cardiomyocytes. These results indicate that the TRPM4 variant, TRPM4 p.I376T, does not alter electrical activity in the murine heart at young ages but decreases the amount of highly glycosylated TRPM4 protein expressed in the heart via an unknown mechanism.

## 1 INTRODUCTION

Transient receptor potential melastatin‐related 4 (TRPM4) is a non‐selective, cation‐permeable channel modulated by transmembrane voltage (Ehara et al., 1988; Launay et al., 2002; Nilius et al., 2003). It is encoded by the mouse *Trpm4* and human *TRPM4* genes, found on chromosome 7 and chromosome 19, respectively (Guinamard et al., 2010; Launay et al., 2002; Nilius et al., 2004).

TRPM4 dysfunction, caused by either gain‐ or loss‐of‐function genetic variants, has been linked to several cardiac conduction disorders, such as cardiac bundle branch block, Brugada syndrome, atrioventricular block and right bundle branch block (Kruse et al., 2009; Liu et al., 2010, 2013; Stallmeyer et al., 2012). In 2016, the progressive familial heart block type I from a large French family was demonstrated to be linked to a point mutation in the human TRPM4 channel at position 376 (TRPM4 p.I376T), leading, in a heterologous expression system, to a gain of expression and function of this channel (Daumy et al., 2016). Although recent in vivo studies have investigated the cardiac consequences of the TRPM4 knockout expression, the cardiac effect related to the TRPM4 gain of expression in cardiac physiology remains to be determined (Arullampalam et al., 2023; Demion et al., 2014; Mathar et al., 2010; 2014, Ozhathil et al., 2021).

To address this question, we generated a *Trpm4* knock‐in mouse harbouring this mutation (*Trpm4* ^I376T/I376T^). With this new *Trpm4* ^I376T/I376T^ mouse model, we investigated whether this TRPM4 p.I376T variant is linked, in vivo, to cardiac dysfunction by assessing the influence of animal age and sex on cardiac electrical activity and by investigating the consequences of this mutation on the TRPM4 protein expression in cardiac tissue.

## 2 MATERIALS AND METHODS

### 2.1 *Trpm4* ^I376T/I376T^ C57BL6/JRj mouse model

In collaboration with the group of Dr Rudy Vennekens, the ingenious targeting laboratory has generated a new *Trpm4* ^I376T/I376T^ mouse model. In brief, a 9.1 kb genomic DNA used to construct the targeting vector was initially subcloned from a positively identified C57BL/6 BAC (Bacterial Artificial Chromosome) clone. The region was designed such that the long homology arm (LA) extends ∼5.65 kb 5′ to the 5′ Lox P cassette, and the short homology arm (SA) extends ∼2.51 kb 3′ to the insertion of the inversion cassette. The inversion cassette is flanked by two mutant Lox sites (Lox 71/66) and consists of the mutant exon 9 (ATC → ACC) and the flanking genomic sequences from upstream of exon 7 to downstream of exon 9 for correct splicing (Inv sdEx7‐9*sa). This cassette was inserted in the reverse direction in intron 9–10. The FRT‐flanked Neo cassette was inserted immediately upstream of the inversion cassette and is 148 bp away from wild‐type exon 9. The targeting region is 914 bp, containing exons 7–9. A SphI restriction enzyme site was engineered in intron 7–8 of the inversion cassette for genotyping purposes (Supporting Information, Figure S1). The *Trpm4* ^I376T/I376T^ (B6JRj.Cg‐TRPM4<I377T>KI) mouse model is a global knock‐in mutation. The corresponding amino acid in its murine orthologue corresponds to TRPM4 p.I377T. Mice were backcrossed onto a C57Bl6/JRj background (Janvier). The C57Bl6/JRj strain has the advantage of being well characterized and readily available from Janvier Laboratories (https://janvier-labs.com/fiche_produit/2-c57bl-6j), enabling us to backcross the mouse model to achieve a genetically homogeneous ‘pure’ background. To fulfil the ‘3R’ criteria (reduce, reuse, refine), male and female *Trpm4* ^I376T/I376T^ mice and wild‐type animals at different matched ages were used for all experiments. Mice were housed in a controlled, specific pathogen‐free environment (21°C ± 1°C; humidity 60%; lights on 06.00–18.00 h; food and water available ad libitum; enriched environment) with a maximum of five mice per cage.

According to the Swiss Federal Animal Protection Law, all animal experiments were performed and approved by Bern's Cantonal Veterinary Administration. This investigation conforms to the *Guide for the Care and Use of Laboratory Animals*, published by the US National Institutes of Health (NIH publication no. 85‐23, revised 1996). For ethical reasons and animal authorization, only young adult mice (≤36 weeks old) were allowed to be investigated. For all experiments using animals, male and female mice have been investigated. For ECG recordings, the age of the animals ranged up to 36 weeks old. For the biochemistry assay, the animals were between 22 and 32 weeks old. For patch‐clamp recordings, the animals were between 27 and 30 weeks old.

### 2.2 Surface ECGs

Three‐lead surface ECGs were recorded for ≥2 min in 12‐, 18‐, 24‐ and 36‐week‐old mice under general anaesthesia (isoflurane, IsofloH, ABBOTT SA, Madrid, Spain; induction, 2.0 vol.% in 1000 cm^3^ O_2_/min; maintenance, 1.5 vol.% in 500 cm^3^ O_2_/min). Body temperature was maintained at 37°C using a thermic pad. Data were collected using an analog‐to‐digital converter (National Instruments, Austin, TX, USA). Power Lab bio‐amplifiers collected the ECG traces, which were low‐pass filtered at 200 Hz and high‐pass filtered at 0.1 Hz, at a sampling rate of 1 kHz. Data from the lead II configuration were analysed offline by LabChart8 Pro (AD Instruments, Castle Hill, NSW, Australia). Initially, the ECG trace was scanned for arrhythmias and noise. Heart rate (HR), P, R–R, P–R and QRS intervals were determined from a stable sequence of ≥10 consecutive sequences (P wave and QRS complex) in each ECG. All parameters were measured by two people in blind conditions (neither individual knew the genotype of the animals).

### 2.3 Western blot

Mice were deeply anaesthetized using a ketamine–xylazine mix (200/20 mg/kg body weight) via intraperitoneal injection. After loss of reflexes, organs were rapidly excised. For the heart and colon, TRPM4 channel expression was assessed in membrane protein lysates using the Mem‐PER Plus Membrane Protein Extraction Kit (Thermo Fisher; ref. 89842). Initially, heart tissue was washed twice in wash solution, cut into four pieces and put into a 2 mL tube (Sarstedt; ref. 72.944.005) containing 10 silica beads (2.8 mm, Milian; ref. 53608) and 20 silica beads (1.4 mm, Milian; ref, 53607) in permeabilization buffer added with Complete^®^ protease inhibitor cocktail (Roche Diagnostics, Mannheim, Germany) to a final concentration of 1×. Homogenization was performed using a Bioprep‐24R device (Allsheng, Hangzhou, China) with the following program: speed, 4260 rpm; linear speed, 7.00 m/s; shaking time, 5 s, followed by a 30 s pause, for six cycles at 4°C. The lysate was transferred into a new 1.5 mL tube, which was then placed on a wheel at 4°C for 15 min. The tube was subsequently centrifuged at 4°C and 16 000*g* for 15 min. The supernatant was discarded, and the pellet was resuspended in solubilization buffer containing Complete^®^ protease inhibitor cocktail (Roche Diagnostics, Mannheim, Germany) to a final concentration of 1×. The sample was returned to the wheel for 1 h at 4°C, then centrifuged at 4°C for 15 min at 16 000*g*. The supernatant was transferred to a new 1.5 mL tube to determine the protein concentration. The lysate sample was measured in triplicate by the Bradford assay and interpolated by a bovine serum albumin standard curve. Samples were denatured at 37°C for 30 min before loading onto a gel. For heart samples with low TRPM4, 100 µg of protein was loaded and run at 200 V for 1 h on 9% polyacrylamide gels. For colon samples with high TRPM4 expression, a non‐saturating loading of 20 µg protein was applied, and the samples were run at 200 V for 1 h on 9% polyacrylamide gels. The Turbo Blot dry blot system (Bio‐Rad, Hercules, CA, USA) was used to transfer the samples to a nitrocellulose membrane. All membranes were stained with Ponceau as a qualitative check for equivalent loading of total protein. The membrane was blocked in Tris Buffer Saline (TBS) 1× containing 5% free‐fat milk overnight at 4°C. The membrane was incubated for 2 h at RT under gentle shaking with rabbit primary anti‐mouse TRPM4 (Q7TN37‐1) antibody (epitope: _2_VGPEKEQSWIPKIFRKKVC_10_; generated by Pineda, Berlin, Germany) and mouse primary anti‐mouse Na^+^/K^+^‐ATPase ab 7671 (α‐subunit; Abcam, Cambridge, UK) diluted 1:500 and 1:1000 in TBS 1× + 0.1% Tween + 0.02% sodium azide, respectively. Membranes were subsequently washed four times for 5 min in TBS 1× + 0.1% Tween before incubating with fluorescent secondary antibodies. Secondary antibodies (IR Dye 800 CW anti‐rabbit and IR Dye 700 CW anti‐mouse, 1:10 000, both in TBS 1× + 0.1% Tween, LI‐COR Biosciences, Lincoln, NE, USA) were added for 1 h under gentle shaking. After three more washes with TBS 1× + 0.1% Tween and two washes in TBS 1× for 5 min each, membranes were scanned with the FUSION FX Spectra^®^ Infrared Imaging System (VILBER smart imaging, Marne‐la‐Vallée, France) to detect fluorescent protein. Subsequent quantitative analysis of protein content was performed by measuring and comparing band densities (equivalent to band fluorescence intensities) using the *Evolution‐Capt software* (VILBER smart imaging, Marne‐la‐Vallée, France). The background was first subtracted from each band (TRPM4 and Na^+^/K^+^‐ATPase), then the TRPM4 band intensity was divided by the Na^+^/K^+^‐ATPase band intensity (for a given sample) and normalized for comparison.

### 2.4 Deglycosylation assays

HEK‐293 cells or homogenized mouse samples were lysed according to the protocol described in the western blot section. One hundred micrograms of these lysates were denatured for 30 min at 37°C in the presence of 1× glycoprotein denaturing buffer and bidistilled H_2_O, to a final volume of 28 µL. Following this incubation, the deglycosylation mix was added [Peptide‐N‐Glycosidase F (PNGase F) mix: 1× G7 buffer: 4 µL, 1× NP40: 4 µL, and 1500 units: 3 µL PNGase F (New England Biolabs, Ipswich, MA, USA)] to the denatured samples and incubated for 1 h at 37°C, 500 rpm. To stop the reaction, 21 µL of 4× NuPAGE sample buffer (Invitrogen, Carlsbad, CA, USA) was added with 100 mM dithiothreitol.

### 2.5 Cardiomyocyte isolation

Mice were deeply anaesthetized using a ketamine–xylazine mix (200/20 mg/kg body weight) via intraperitoneal injection. After loss of reflexes, freshly isolated adult murine ventricular cardiomyocytes were isolated using a procedure like the one previously published by Ackers‐Johnson et al. (2016), without the addition of 2,3‐butanedione monoxime.

### 2.6 Cellular electrophysiology

The ‘TRPM4 current’ was recorded in the whole‐cell configuration at room temperature (22°C–23°C) using a VE‐2 amplifier (Alembic Instruments, USA). Borosilicate glass pipettes were pulled to a series resistance of ∼2 MΩ. The pClamp software, v.8 (Axon Instruments, Union City, CA, USA) was used for recordings. Data were analysed using pClamp software, v.8 (Axon Instruments) and OriginPro, v.7.5 (OriginLab Corp., Northampton, MA, USA). Similar approaches to the one used by Vandewiele et al. (2022) have been used to record ‘TRPM4 current’. In brief, the voltage protocol consists of a 2 s depolarization to +10 mV to induce Ca^2+^ overload in the sarcoplasmic reticulum (SR), followed by a 10 s depolarization to −50 mV. To ensure proper loading of the SR with Ca^2+^, this protocol was preceded by a 10 ms depolarization to +10 mV at 1 Hz, repeated 10 times. The holding potential was −80 mV. Currents were sampled at 5 kHz. The extracellular solution contained (mM): 117 NaCl, 20 CsCl, 10 HEPES, 10 glucose, 2 CaCl_2_, 1.8 MgCl_2_ and 0.01 isoprenaline (pH adjusted to 7.40 with CsOH). The pipette solution contained (mM): 50 caesium aspartate, 60 CsCl, 5 Na_2_ATP, 10 HEPES, 0.05 EGTA, 0.0215 CaCl_2_ and 1 MgCl_2_ (pH adjusted to7.20 with CsOH). The ‘TRPM4 current’ was quantified by calculating the difference in current between the end and the beginning of the current recorded during the 10 s depolarization pulse to −50 mV. Only cardiac cells with a seal resistance of >4 GΩ were included in the analysis.

### 2.7 Data analysis and statistics

**FIGURE 1:**
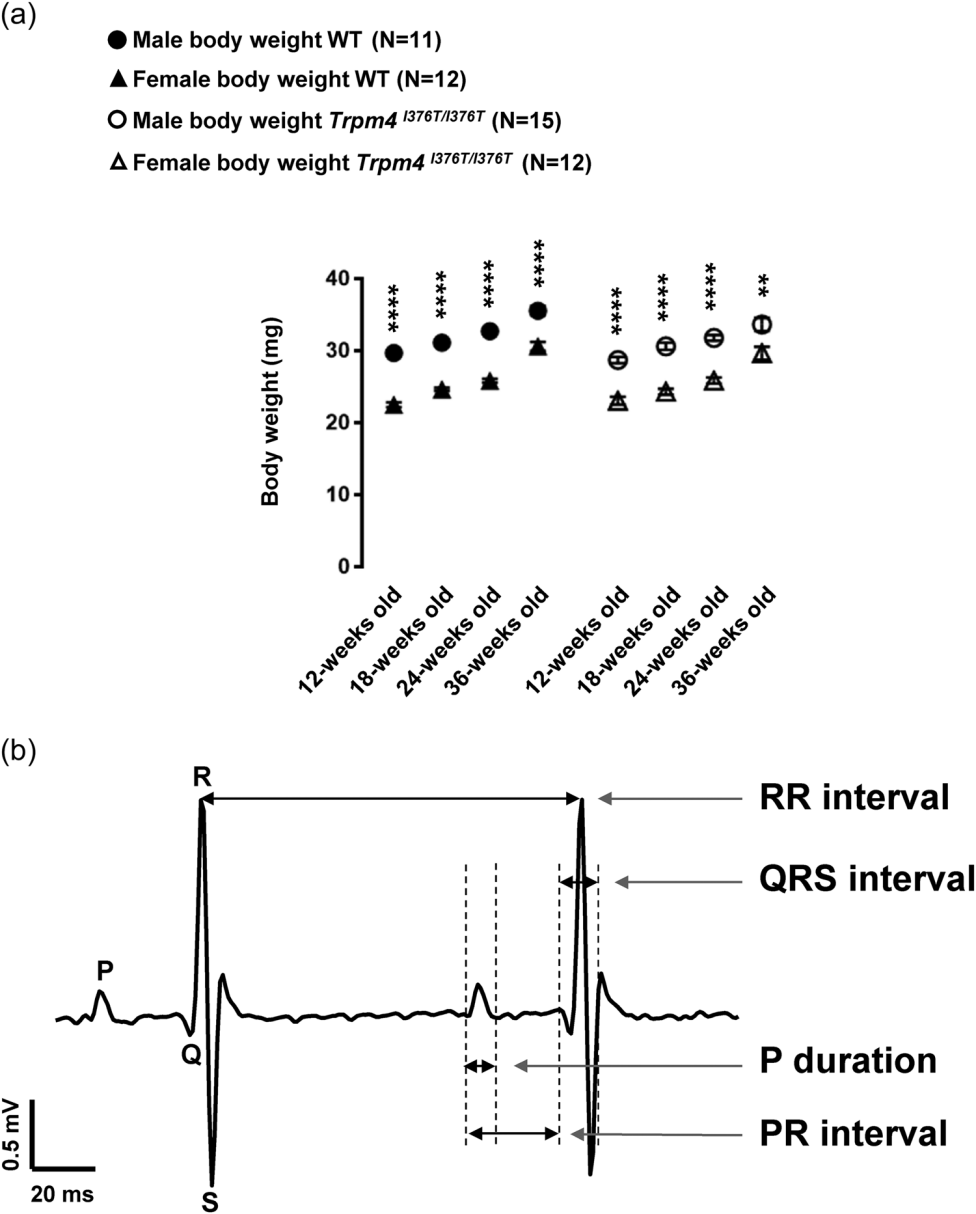
Body weights of wild‐type and *Trpm4* ^I376T/I376T^ mice at different ages and ECG parameters. (a) Male and female body weights at 12, 18, 24 and 36 weeks of age for wild‐type and *Trpm4* ^I376T/I376T^ mice. **P ≤ 0.01 and ****P ≤ 0.0001 (*N* ≥ 11 per group). An unpaired non‐parametric *t*‐test followed by a *post hoc* Mann–Whitney *U*‐test was used to compare male and female groups from the same genotype. (b) Representative ECG trace from an adult male wild‐type mouse and the different parameters investigated.

Data are represented as means ± SEM. Statistical analyses were performed using Prism 7 GraphPad software (GraphPad by Dotmatics, San Diego, CA, USA). An unpaired non‐parametric *t*‐test was used to compare two unpaired groups (Figure 8c). An unpaired non‐parametric *t*‐test followed by a *post hoc* Mann–Whitney *U*‐test was used to compare two unpaired groups (Figure 1). A Column statistic test was used to compare two unpaired groups by performing a D'Agostino–Pearson normality test followed by a Wilcoxon signed‐rank test with a theoretical median of 100 (Figures 4a, b, 5a, b, 7b and 8b). A value of *P* < 0.05 was considered significant. No multi‐group comparison was made in this study. For Figure 1, ‘*N*’ corresponds to the number of animals weighed. For Figures 4a, b, 5a, b, 6c and 7b, ‘*n*’ corresponds to the number of samples quantified and ‘*N*’ to the number of animals used. For Figure 8a, b, ‘*n*’ corresponds to the number of ventricular cardiomyocytes patched and ‘*N*’ to the number of animals used. For Table 1, ‘*N*’ corresponds to the number of animals used.

**TABLE 1:** Evolution of the ECG parameters in male and female wild‐type and *Trpm4* ^I376T/I376T^ mice at 12, 18, 24 and 36 weeks of age

Mouse genotype and sex		R–R interval (ms)			
		12th week	18th week	24th week	36th week
Wild‐type	♂	131.2 ± 2.9 (*N* = 11)	123.9 ± 2.8 (*N* = 11)	134.2 ± 2.2 (*N* = 11)	129.0 ± 3.9 (*N* = 11)
*Trpm4* ^I376T/I376T^		126.0 ± 2.8 (*N* = 16)	127.1 ± 3.4 (*N* = 16)	123.7 ± 3.3 (*N* = 16)	133.6 ± 3.3 (*N* = 16)
Wild‐type	♀	137.7 ± 1.9 (*N* = 12)	131.5 ± 3.2 (*N* = 12)	136.5 ± 3.3 (*N* = 12)	122.3 ± 3.7 (*N* = 12)
*Trpm4* ^I376T/I376T^		130.9 ± 1.6 (*N* = 12)	133.5 ± 3.1 (*N* = 12)	127.4 ± 2.0 (*N* = 12)	126.3 ± 2.7 (*N* = 12)

		R–R interval (ms)			
		12th week	18th week	24th week	36th week
Wild‐type	♂	40.6 ± 0.7 (*N* = 11)	40.6 ± 0.7 (*N* = 11)	40.6 ± 0.8 (*N* = 11)	40.7 ± 0.9 (*N* = 11)
*Trpm4* ^I376T/I376T^		40.3 ± 0.7 (*N* = 16)	40.9 ± 0.7 (*N* = 16)	39.1 ± 0.8 (*N* = 16)	39.2 ± 0.9 (*N* = 16)
Wild‐type	♀	42.5 ± 0.6 (*N* = 12)	41.2 ± 0.9 (*N* = 12)	41.1 ± 1.0 (*N* = 12)	40.8 ± 0.7 (*N* = 12)
*Trpm4* ^I376T/I376T^		41.8 ± 0.5 (*N* = 12)	43.1 ± 0.8 (*N* = 12)	41.9 ± 0.5 (*N* = 12)	41.0 ± 0.7 (*N* = 12)

		QRS interval (ms)			
		12th week	18th week	24th week	36th week
Wild‐type	♂	10.6 ± 0.1 (*N* = 11)	10.7 ± 0.2 (*N* = 11)	10.4 ± 0.2 (*N* = 11)	10.6 ± 0.2 (*N* = 11)
*Trpm4* ^I376T/I376T^		10.5 ± 0.4 (*N* = 16)	10.8 ± 0.3 (*N* = 16)	10.6 ± 0.3 (*N* = 16)	10.5 ± 0.2 (*N* = 16)
Wild‐type	♀	10.9 ± 0.3 (*N* = 12)	11.1 ± 0.2 (*N* = 12)	10.8 ± 0.4 (*N* = 12)	10.9 ± 0.2 (*N* = 12)
*Trpm4* ^I376T/I376T^		10.5 ± 0.2 (*N* = 12)	10.6 ± 0.2 (*N* = 12)	10.7 ± 0.2 (*N* = 12)	10.6 ± 0.3 (*N* = 12)

		P duration (ms)			
		12th week	18th week	24th week	36th week
Wild‐type	♂	28.9 ± 0.9 (*N* = 11)	32.3 ± 1.0 (*N* = 11)	27.0 ± 1.9 (*N* = 11)	26.3 ± 2.7 (*N* = 11)
*Trpm4* ^I376T/I376T^		29.0 ± 0.8 (*N* = 16)	31.4 ± 0.6 (*N* = 16)	26.7 ± 1.6 (*N* = 16)	24.3 ± 2.0 (*N* = 16)
Wild‐type	♀	33.7 ± 0.6 (*N* = 12)	32.0 ± 0.6 (*N* = 12)	31.2 ± 1.3 (*N* = 12)	28.0 ± 2.3 (*N* = 12)
*Trpm4* ^I376T/I376T^		29.8 ± 0.6 (*N* = 12)	32.4 ± 1.0 (*N* = 12)	29.6 ± 2.0 (*N* = 12)	29.8 ± 0.6 (*N* = 12)

		Heart rate (beats/min)	
		12th week	18th week	24th week	36th week
Wild‐type	♂	459 ± 10 (*N* = 11)	487 ± 11 (*N* = 11)	448 ± 8 (*N* = 11)	469 ± 13 (*N* = 10)
*Trpm4* ^I376T/I376T^		480 ± 12 (*N* = 16)	477 ± 13 (*N* = 16)	490 ± 13 (*N* = 15)	453 ± 11 (*N* = 15)
Wild‐type	♀	437 ± 6 (*N* = 12)	459 ± 11 (*N* = 12)	442 ± 11 (*N* = 12)	495 ± 14 (*N* = 12)
*Trpm4* ^I376T/I376T^		459 ± 6 (*N* = 12)	453 ± 12 (*N* = 12)	472 ± 8 (*N* = 12)	478 ± 10 (*N* = 12)

## 3 RESULTS

The role of TRPM4 in cardiac function has been investigated using a newly generated *Trpm4* ^I376T/I376T^ mouse line, in which the codon ATC (isoleucine/I_377_) in exon 9 of *Trpm4* has been mutated to ACC (threonine/T_377_) (Supporting Information, Figure S1). In the homozygous state, the *Trpm4* ^I376T/I376T^ mouse line investigated here showed no increase in mortality or alteration in Mendelian genetic transmission. The body weights of wild‐type and *Trpm4* ^I376T/I376T^ mice were similar at the different ages (12, 18, 24 and 36 weeks old; Figure 1a), regardless of the sex of the animal. However, as expected, the body weights of female animals were always lighter than those of male mice (Figure 1a).

### 3.1 The *Trpm4* ^I376T/I376T^ knock‐in did not affect surface ECG parameters in adult mice compared with control animals

Surface ECGs were recorded to assess the cardiac electrophysiological parameters depicted in Figure 1b of four groups of mice (male and female wild‐type, and male and female *Trpm4* ^I376T/I376T^) at different ages (12, 18, 24 and 36 weeks old; Figure 1b). These ECGs showed no differences in the measured parameters (R–R interval, P duration, P–R interval, QRS interval and heart rate; Table 1). Given the absence of ECG alterations, independent of animal sex, the following experiments (biochemistry assays and whole‐cell patch‐clamp recordings) were performed in both sexes, and the data have been pooled.

### 3.2 TRPM4 expression is downregulated in the *Trpm4* ^I376T/I376T^ mouse heart

**FIGURE 2:**
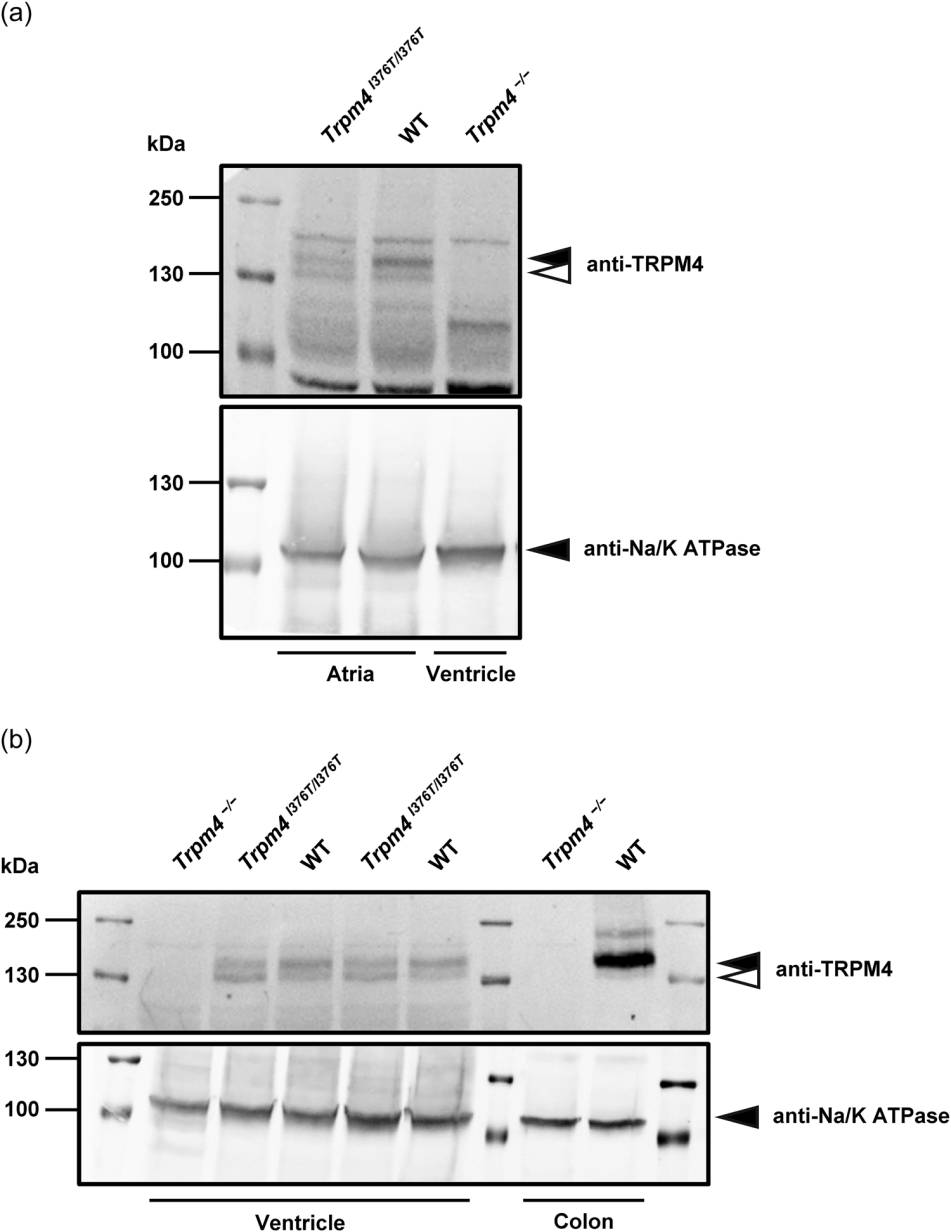
TRPM4 protein expression in membrane preparation from mouse hearts. (a) Western blot from wild‐type, *Trpm4* ^I376T/I376T^ atria and *Trpm4* ^−/−^ mouse ventricle. (b) Western blot from wild‐type, *Trpm4* ^I376T/I376T^ and *Trpm4* ^−/−^ mouse ventricles and colon. For TRPM4 blots, the two triangles highlight the highly glycosylated TRPM4 form (HG‐TRPM4, black triangle) and the less or core‐glycosylated form (CG‐TRPM4, white triangle).

**FIGURE 3:**
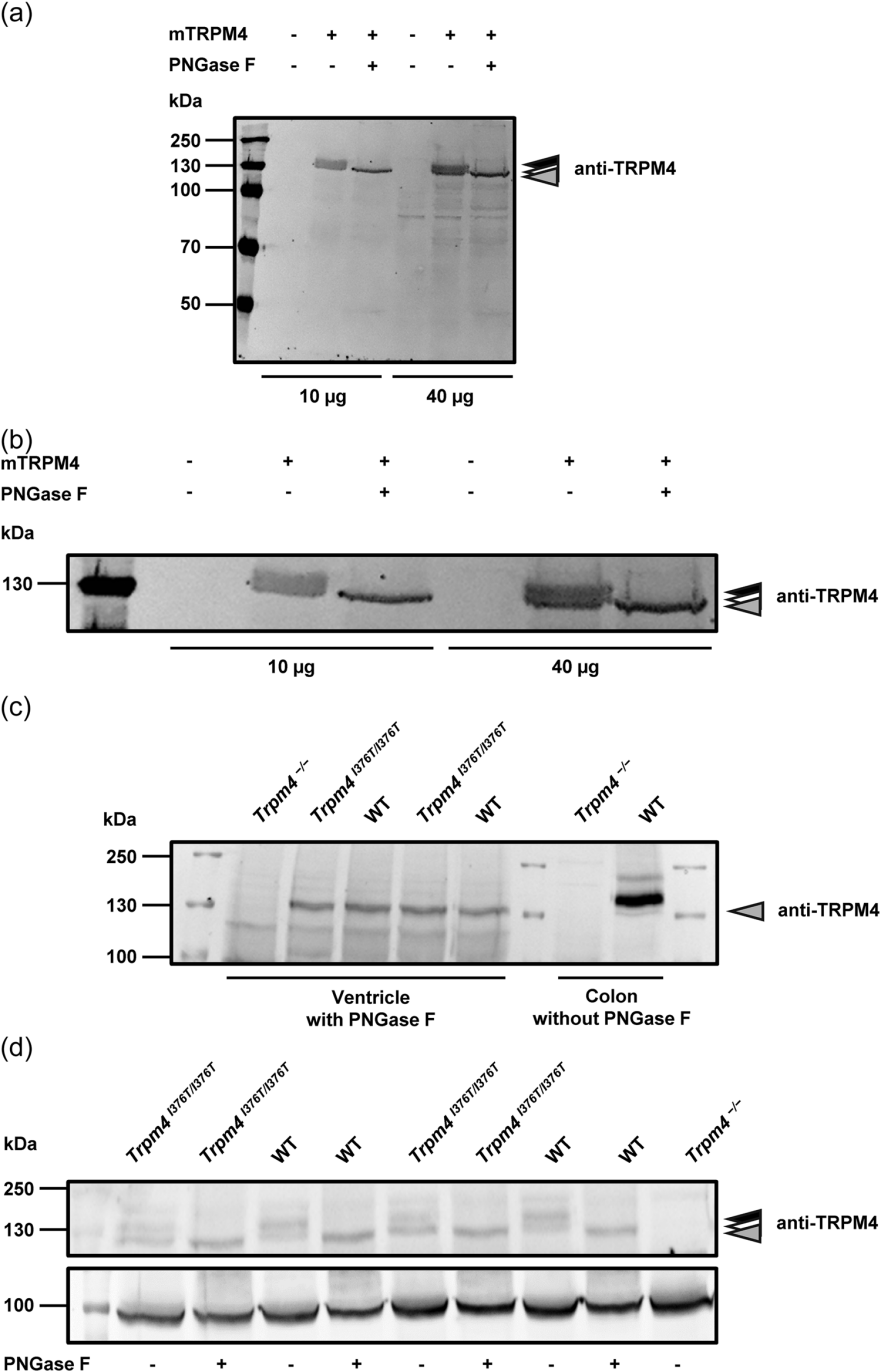
TRPM4 deglycosylation. (a) Western blot from a heterologous expression system overexpressing mouse TRPM4 treated (+) or not (−) with the deglycosylase enzyme PNGase F, showing the modification of the highly glycosylated TRPM4 form (HG‐TRPM4, black triangle) and the less or core‐glycosylated form (CG‐TRPM4, white triangle) molecular weight after treatment (grey triangle). (b) Magnification of (a). (c, d) Western blot from membrane preparations from wild‐type, *Trpm4* ^I376T/I376T^ and *Trpm4* ^−/−^ mouse ventricles after treatment with the enzyme PNGase F, showing the presence of the deglycosylated form of TRPM4 (grey triangle). Colon samples without treatment has been added to identified the glycosylated band of TRPM4 protein. In (a) and (b), the labels 10 and 40 µg refer to the total amount of protein loaded onto the western blots.

**FIGURE 4:**
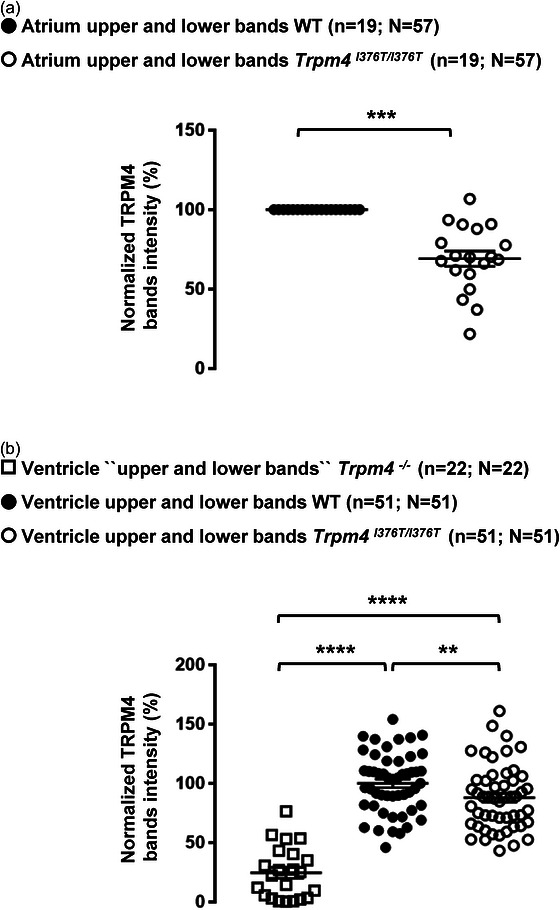
Western blot quantification of TRPM4 membrane protein from atria and ventricles. (a) Dot blots showing the normalized TRPM4 upper and lower band intensities from wild‐type and *Trpm4* ^I376T/I376T^ atria. (b) Dot blots showing the normalized TRPM4 upper and lower band intensities from wild‐type, *Trpm4* ^I376T/I376T^ and *Trpm4* ^−/−^ ventricles. **P ≤ 0.01, ***P ≤ 0.001 and ****P ≤ 0.0001 (*n* ≥ 19 per group). A column statistic test was used to compare two unpaired groups by performing a D'Agostino–Pearson normality test followed by a Wilcoxon signed‐rank test with a theoretical median of 100 (a and b).

We investigated the expression pattern of the TRPM4 protein using a membrane preparation of cardiac tissue. In comparison to the knockout sample for TRPM4 (*Trpm4* ^−/−^), the wild‐type and the new *Trpm4* ^I376T/I376T^ mouse model express TRPM4 protein in atria and ventricles (Figure 2a, b). The typical ’TRPM4 doublet’ is clearly observable in atrial and ventricular lysates (Figure 2a, b). As previously reported, this doublet is observed in heterologous expression systems and human cardiac samples owing to the presence of a mature, highly glycosylated TRPM4 channel, also named highly glycosylated‐TRPM4 (HG‐TRPM4; upper band/black triangle) and a partly or non‐glycosylated band, also called core glycosylated‐TRPM4 (CG‐TRPM4; lower band/white triangle) (Figures 2 and 3) (Syam et al., 2014; Woo et al., 2013). To confirm the glycosylation pattern of this doublet in cardiac samples, we initially tested the effect of deglycosylation with PNGase F in HEK‐293 cells as a control, then applied the same treatment to cardiac samples. As shown in Figure 3, in HEK‐293 cells, the overexpression of mouse TRPM4 results in a doublet in the absence of treatment (Figure 3a, b). However, treatment of the transfected cell lysates with the enzyme PNGase F removes the glycosylation of TRPM4, resulting in a single band with a molecular weight lower than the two untreated bands (grey triangle), confirming the presence of both the highly glycosylated and core glycosylated forms of TRPM4 in non‐treated transfected cells (Figure 3a, b). Similar experiments using ventricular samples showed a single TRPM4 band after deglycosylation in both genotypes, suggesting that the upper band in the untreated samples corresponds to the highly glycosylated fraction (Figure 3c, d). Moreover, Figure 3d suggests that the lower band corresponds to the core glycosylated form of TRPM4, as observed in the heterologous expression system (Figure 3d). As presented in Figure 4, the quantification of the lower and upper bands, together, in atria and ventricles shows a decrease in TRPM4 expression in *Trpm4* ^I376T/I376T^ mouse hearts compared with wild‐type mouse hearts. This effect was more pronounced in atria than in ventricles (Figure 4a, b).

**FIGURE 5:**
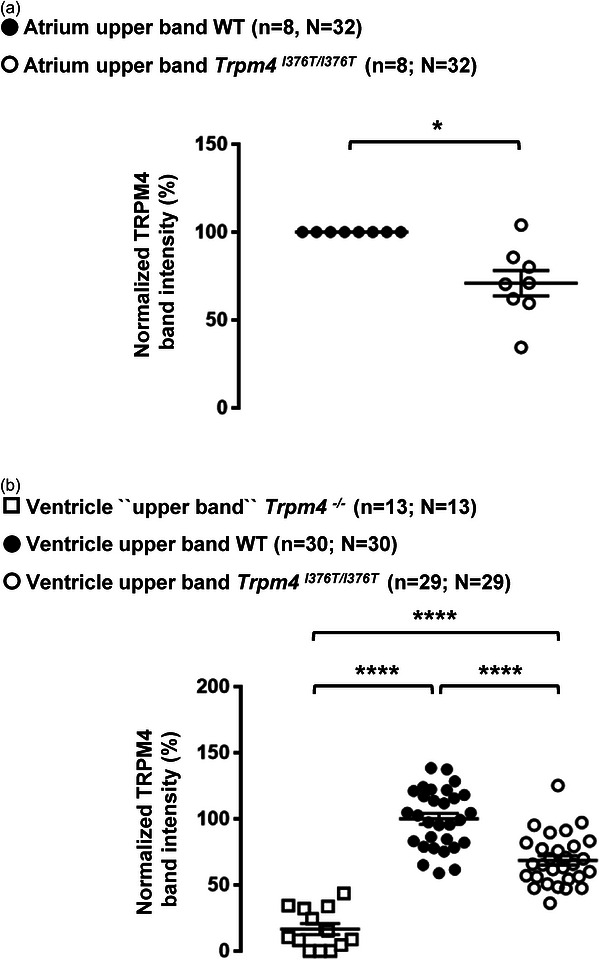
Western blot quantification of upper TRPM4 membrane protein from atria and ventricles. (a) Dot blots showing the normalized TRPM4 upper band intensities from wild‐type and *Trpm4* ^I376T/I376T^ atria. (b) Dot blots showing the normalized TRPM4 upper band intensities from wild‐type, *Trpm4* ^I376T/I376T^ and *Trpm4* ^−/−^ ventricles. *P ≤ 0.05 and *** *P* ≤ 0.001 (*n* ≥ 13 per group). A column statistic test was used to compare two unpaired groups by performing a D'Agostino–Pearson normality test followed by a Wilcoxon signed‐rank test with a theoretical median of 100 (a and b).

Unexpectedly, compared with wild‐type cardiac lysates, the knock‐in tissues exhibited a reduction in the upper band intensity (Figures 2b and 3d). Based on these observations, upper and lower bands from atria and ventricles of both genotypes were quantified separately. For both genotypes, the atrium upper band is significantly more intense than the atrium lower band, indicating the presence of more highly glycosylated TRPM4 channels than the core glycosylated form (percentage of decrease of wild‐type atrium lower band intensity compared with wild‐type atrium upper band intensity: −34.2% ± 7.2%; *n* = 8 samples and *N* = 32 animals; *p* ⩽0.01 by performing a D'Agostino and Pearson normality test followed by a Wilcoxon Signed‐Rank test with a theoretical median of 100 and percentage of decrease of *Trpm4* ^I376T/I376T^ atrium lower band intensity compared to *Trpm4* ^I376T/I376T^ atrium upper band intensity: −22.1% ± 4.8%; *n* = 8 samples and *N* = 32 animals; *P* ≤ 0.01 by performing a D'Agostino–Pearson normality test followed by a Wilcoxon signed‐rank test with a theoretical median of 100). Moreover, comparing either the upper atrial bands between the two genotypes or the lower atrial bands between the two genotypes revealed a significant decrease only in the upper atrial bands from *Trpm4* ^I376T/I376T^ mice compared with wild‐type animals, indicating decreased highly glycosylated mutant TRPM4 channels compared with wild‐type TRPM4 channels (Figure 5a). Similar quantifications with ventricle samples also indicate that the highly glycosylated wild‐type TRPM4 channel is significantly more present than the core glycosylated form (percentage of decrease of wild‐type ventricle lower band intensity compared with wild‐type ventricle upper band intensity: −18.0% ± 5.2%; *n* = 30 samples and *N* = 30 animals; *P* ≤ 0.01 by performing a D'Agostino–Pearson normality test followed by a Wilcoxon signed‐rank test with a theoretical median of 100). However, in the *Trpm4* ^I376T/I376T^ ventricle, the highly glycosylated band is less intense than the core glycosylated band, suggesting that more core glycosylated TRPM4 mutant channels are present in membrane preparations than their highly glycosylated form (percentage of increase of *Trpm4* ^I376T/I376T^ ventricle lower band intensity compared with *Trpm4* ^I376T/I376T^ ventricle upper band intensity: +30.4% ± 6.1%; *n* = 30 samples and *N* = 30 animals; *P* ≤ 0.001 by performing a D'Agostino–Pearson normality test followed by a Wilcoxon signed‐rank test with a theoretical median of 100). Moreover, comparison of the ventricle upper bands intensities between wild‐type and mutant animals and lower band intensities between wild‐type and mutant animals showed a decrease, compared with wild‐type, only in highly glycosylated TRPM4 mutant channels (upper band) (Figure 5b).

### 3.3 TRPM4 expression is not altered in the *Trpm4* ^I376T/I376T^ mouse colon

**FIGURE 6:**
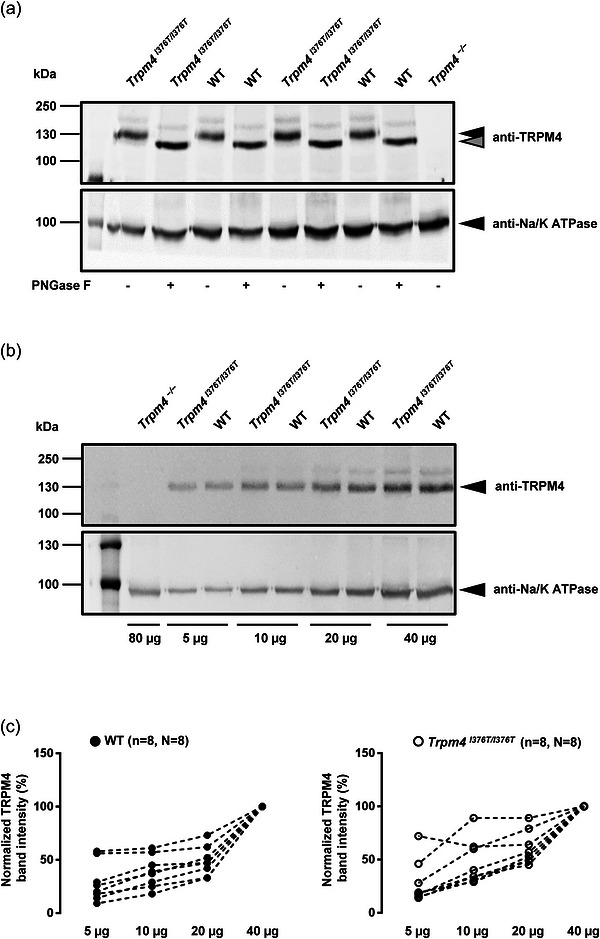
TRPM4 deglycosylation from wild‐type and *Trpm4* ^I376T/I376T^ colons. (A) Western blot from wild‐type and *Trpm4* ^I376T/I376T^ colons treated (+) or not (−) with the deglycosylase enzyme PNGase F, showing the modification of the highly glycosylated TRPM4 form (HG‐TRPM4, black triangle), after treatment (grey triangle). (b) Western blot showing the increased intensity of the signal corresponding to TRPM4 protein expression in wild‐type and *Trpm4* ^I376T/I376T^ colon, depending on the amount of protein loaded. (c) Dot blots showing the normalized TRPM4 band intensities from wild‐type and *Trpm4* ^I376T/I376T^ colon, highlighting the absence of saturating signal for the TRPM4 band with 20 µg of protein (*n* = 8 per group).

**FIGURE 7:**
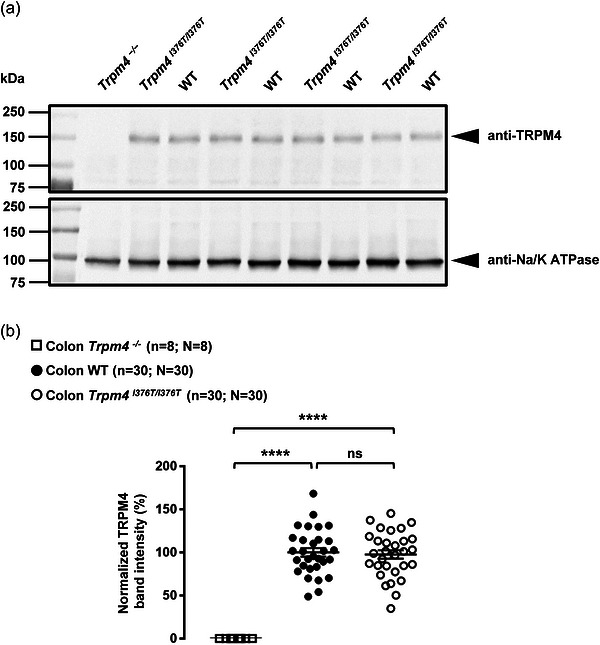
Western blot and quantification of the TRPM4 membrane protein expressed in the colon from wild‐type and *Trpm4* ^I376T/I376T^ mice. Western blot (a) and quantification (b) of TRPM4 expressed in colon from the two genotypes, suggesting no difference. ns, non‐significant; ****P ≤ 0.0001 (*n* ≥ 8 per group). In (b), a column statistic test was used to compare two unpaired groups by performing a D'Agostino–Pearson normality test followed by a Wilcoxon signed‐rank test with a theoretical median of 100.

The unexpected decrease in the highly glycosylated bands observed in cardiac tissue from the new *Trpm4* ^I376T/I376T^ mouse model, compared with wild‐type mouse hearts, prompted us to investigate TRPM4 expression in other tissues. We used mouse colons, in which TRPM4 expression is observed by western blot as a single band corresponding to the highly glycosylated form of TRPM4, similar to the upper band in the heart (Figures 2b and 3c) (Woo et al., 2013). First, we confirmed that the TRPM4 band observed in wild‐type and knock‐in colons is glycosylated by performing deglycosylation experiments similar to those with cardiac lysates (Figure 6a). Second, because TRPM4 signal intensity is higher in the colon than in the heart, varying amounts of protein from wild‐type and *Trpm4* ^I376T/I376T^ colons were loaded to estimate the amount required for proper quantification within the non‐saturating range. As shown in Figure 6, the increase in the TRPM4 band intensity is correlated with the rise in the amount of protein loaded into the gel (Figure 6b, c). Moreover, the quantification presented in Figure 6c suggests that the signal for 20 µg of protein is non‐saturating and can be used for further comparison between the two genotypes (Figure 6c). The quantification of TRPM4 band intensities in the colon using 20 µg of protein showed no difference between the two genotypes (Figure 7a, b). Overall, these data suggest that the mechanism underlying the decrease in the highly glycosylated TRPM4 variant protein is organ specific.

### 3.4 The ‘TRPM4 current densities’ are not altered in the *Trpm4* ^I376T/I376T^ ventricular cardiomyocytes

**FIGURE 8:**
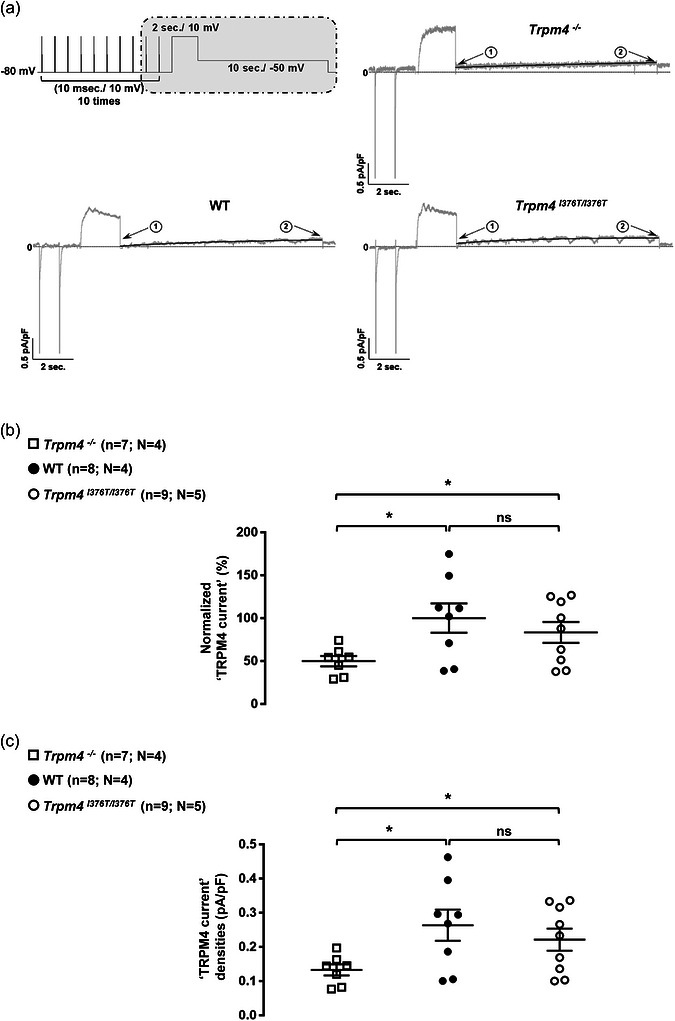
‘TRPM4 current’ quantification. (a) Raw traces of ‘TRPM4 currents’ recorded in the different genotypes. The upper left panel shows the protocol applied, and only the traces within the grey area are shown in the other panels. The numbers 1 and 2 indicate where the current densities were measured at the beginning and end of the 10 s pulse at −50 mV. (b) Dot blots showing the normalized ‘TRPM4 current’ from wild‐type, *Trpm4* ^I376T/I376T^ and *Trpm4* ^−/−^ adult ventricular cardiomyocytes. (c) Dot blots showing the current densities of ‘TRPM4 current’ from wild‐type, *Trpm4* ^I376T/I376T^ and *Trpm4* ^−/−^ adult ventricular cardiomyocytes. ns, non‐significant; *P ≤ 0.05 (*n* ≥ 7 per group). A column statistic test was used to compare two unpaired groups by performing a D'Agostino–Pearson normality test, followed by a Wilcoxon signed‐rank test with a theoretical median of 100 (b), and an unpaired non‐parametric *t*‐test was used to compare two unpaired groups (c).

Syam et al. (2014) observed that tunicamycin‐mediated deglycosylation of TRPM4 channels in HEK293 cells increased the TRPM4 current compared with the non‐treated condition. This motivated us to investigate, using a protocol similar to that published by Vandewiele et al. (2022), the ‘TRPM4 current’ in freshly isolated adult murine ventricular cardiomyocytes from *Trpm4* ^−/−^, wild‐type and *Trpm4* ^I376T/I376T^ mouse lines. As shown in Figure 8, although these ‘TRPM4 current densities’ are extremely small in our experimental conditions, the ‘TRPM4 current’ is significantly decreased in *Trpm4* ^−/−^ cells compared with both wild‐type and *Trpm4* ^I376T/I376T^ cardiomyocytes (Figure 8a–c). However, no statistical differences were observed between wild‐type and *Trpm4* ^I376T/I376T^, indicating that the reduction of glycosylated TRPM4 channels of *Trpm4* ^I376T/I376T^ cardiac cells does not alter the ‘TRPM4 current densities’ (Figure 8b, c).

## 4 DISCUSSION

The main results of this study are as follows: (1) homozygous knock‐in mice carrying the TRPM4 p.I376T variant leading to striking cardiac conduction alterations in humans do not present observable ECG alterations, at least until 36 weeks of age; (2) the highly glycosylated fraction of TRPM4 protein in atrial and ventricular tissues is reduced in knock‐in hearts; and (3) at the cellular level, no alteration of the TRPM4 current was observed, whereas a clear reduction was recorded in knock‐out cardiomyocytes.

Clinical studies unequivocally demonstrated the role of many *TRPM4* genetic variants in cardiac conduction disorders (Kecskes et al., 2015; Liu et al., 2013; Palladino et al., 2022; Stallmeyer et al., 2012). In addition, in the past, *Trpm4* ^−/−^ knockout mouse models have been generated to decipher the role of TRPM4 in cardiac physiology and pathophysiology (Arullampalam et al., 2023; Demion et al., 2014; Mathar et al., 2010, 2014; Ozhathil et al., 2021). However, these investigations yielded inconsistent observations across the different *Trpm4* ^−/−^ mouse lines. Knowing that many *TRPM4* variants not only cause loss of function but also gain of function, we decided to generate a knock‐in p.I376T TRPM4 mouse line (*Trpm4* ^I376T/I376T^), corresponding to p.I377T in humans. This variant was linked to atrioventricular block in a large French pedigree and was reported to lead to a gain of expression and a gain of function using a heterologous cellular expression system (Daumy et al., 2016). Moreover, to compare data collected from the knock‐in line with our in‐house *Trpm4* ^−/−^ mouse model, a new *Trpm4* ^I376T/I376T^ mouse strain has been generated on a similar pure C57BL6/JRj genetic background.

In contrast to the absence of any electrical disturbance, biochemical investigations revealed that the TRPM4 protein from *Trpm4* ^I376T/I376T^ hearts exhibits an overall decrease of TRPM4 protein present at the membrane compared with wild‐type animals and, more specifically, the highly glycosylated form of the protein. Surprisingly, the diminution of this glycosylated form, in *Trpm4* ^I376T/I376T^ hearts, which is supposed to be responsible for the generation of the TRPM4 current, does not affect the ‘TRPM4 current’ recorded in freshly isolated cardiomyocytes of those mice compared with wild‐type cardiac cells. One explanation for this observation might be attributable to a compensatory mechanism between the decrease of highly glycosylated TRPM4 mutant channel at the plasma membrane, which should lead to the reduction of the ‘TRPM4 current’, and a gain of function of this mutant channel, as already reported (Daumy et al., 2016).

Protein glycosylation is a complex co‐ and post‐translational process that involves the addition of a carbohydrate (a polymer of sugars) to specific amino acid residues (He et al., 2024). The main glycosylation processes are *O*‐glycosylation, in which the glycan moieties are attached to the hydroxyl group of serine, threonine or tyrosine, and *N*‐glycosylation, in which the glycan chain is attached to the nitrogen group of asparagine or arginine. We reported that the TRPM4 channel is *N*‐glycosylated via the addition of glycans on the asparagine at position 992 in the human isoform (Syam et al., 2014; Woo et al., 2013). As reported in those papers and other investigations, biochemical experiments using overexpression systems reveal at least two glycosylated forms of the TRPM4 channel at the cell surface: the highly glycosylated form and the core glycosylated form (Syam et al., 2014; Woo et al., 2013). Interestingly, although glycosylation of the TRPM4 protein does not appear to influence the function of the channel or its forward trafficking significantly, this modification increases the stability of the highly glycosylated TRPM4 channel at the cell surface by decreasing its internalization (Woo et al., 2013). The fact that the highly glycosylated TRPM4 channel is less present in the membrane preparation of *Trpm4* ^I376T/I376T^ mouse heart compared with wild‐type cardiac tissue might be explained either by a modification of the *N*‐glycosylation pattern (the mutant TRPM4 channel is glycosylated but in a different way from the wild‐type TRPM4 channel), not detected via our approaches, leading to either a decrease in the stability of the TRPM4 pathological mutant at the cell surface or a degradation of the misfolded mutant TRPM4 protein owing this modified pattern, or both.

Knowing that the glycosylation profile of proteins is modified in the murine heart with ageing, it is tempting to propose that despite the absence of specific electrical disturbances for the *Trpm4* ^I376T/I376T^ mouse at a young adult age (∼30 weeks old in mice, corresponding to 20–30 years in humans), at an older age, related to a differential modification type of the glycosylation pattern of the TRPM4 p.I376T channel compared with the wild‐type TRPM4 channel, the ECG parameters will be altered between knock‐in and wild‐type animals (Franzka et al., 2021). Such an age‐dependent modification of the glycosylation profile might increase the stability of the TRPM4 p.I376T channel at the cell surface relative to wild‐type channels, thereby compensating for the mutation‐induced channel degradation. This mechanism would be expected to reduce the difference in TRPM4 channel expression at the cell surface between the two genotypes, leading, overall, to an increase in the ‘TRPM4 current’ recorded in aged *Trpm4* ^I376T/I376T^ cardiac cells and a perturbation of the ECG, as observed in adult patients.

Although the expression of the TRPM4 channel mutant is not organ specific, the absence of a decrease in the highly glycosylated TRPM4 channel in *Trpm4* ^I376T/I376T^ colon indicates cardiac‐specific mechanisms underlying this alteration. Moreover, knowing that the expression of the Na^+^/K^+^‐ATPase α‐subunit at the plasma membrane depends on the *N*‐glycosylation of the chaperone Na^+^/K^+^‐ATPase β2‐subunits, the similar expression of Na^+^/K^+^‐ATPase α‐subunit in ventricles from both genotypes suggests that the effect observed on the TRPM4 channel in the heart is not a general mechanism for all *N*‐glycosylated cardiac proteins (Tokhtaeva et al., 2010). As recently published, the *N*‐glycome profile of proteins is organ specific, probably owing to tissue‐dependent expression of enzymes involved in the *N*‐glycosylation pathway (Chrysinas et al., 2024; Helm et al., 2024). Based on these observations, it is tempting to propose that: (1) the *N*‐glycosylation process and profile of TRPM4 channels differ between the heart and the colon; and (2) the presence, in the TRPM4 protein, of the point mutation I376 in humans (377 in mice) might affect those parameters mainly for the cardiac TRPM4 channel.

The unexpected decrease in the TRPM4 p.I376T protein at the membrane of cardiac tissue, compared with the increase observed in the heterologous expression system (increase or no alteration of expression of TRPM4 p.I376T compared with wild‐type channel), raises questions about the choice of using such an overexpression system in investigating TRPM4 variants (Daumy et al., 2016). Based on these in vitro approaches, we and others have concluded that gains and losses of expression and function might ultimately lead to similar cardiac dysfunction. Although this observation is unusual, the phenomenon has been proposed to result from a molecular mechanism similar to that described for supra‐normal conduction (Abriel et al., 2012). Based on the data presented in this manuscript, it is essential to consider that loss‐ and gain‐of‐function descriptions of TRPM4 pathological variants in a heterologous expression system might reflect only one of these dysfunctions in physiological conditions, owing to complex, yet unknown, regulatory mechanisms, such as *N*‐glycosylation of the channel. Moreover, we must be careful with both cellular and mouse models, and it might be that induced pluripotent stem cell‐derived cardiomyocytes from patients could be a better approach to elucidate the consequences of variants of the TRPM4 protein present in the human population.

### 4.1 Limitations

Among the limitations of this study, it is worth noting that TRPM4 is widely expressed across organs and tissues, including the brain and endocrine system. The observed alteration in TRPM4 glycosylation might be attributable to indirect mechanisms. Moreover, the use of PNGase F to completely trim the carbohydrate linked to the asparagine residue of the TRPM4 channel hinders a detailed understanding of the glycosylation pattern of the channel and its potential modification in the genetically modified mouse line *Trpm4* ^I376T/I376T^.

## 5 CONCLUSION

In conclusion, investigations of the newly generated *Trpm4* ^I376T/I376T^ mouse line at the young adult age could not fully elucidate the molecular or cellular mechanisms underlying the alterations in cardiac conduction observed in humans carrying this pathogenic variant. Unexpectedly, however, it demonstrated significant modifications in the glycosylation of the TRPM4 protein in atrial and ventricular tissues. The consequences of the biochemical alterations for the pathological phenotype observed in humans remain to be explored.

## AUTHOR CONTRIBUTIONS

Jean‐Sébastien Rougier conceived and designed the experiments. Sabrina Guichard, Emanuele Di Lorenzo, Dominic Schneiter, Maria Essers, Prakash Arullampalam and Jean‐Sébastien Rougier collected, analysed and interpreted the data. Jean‐Sébastien Rougier drafted the manuscript. Jean‐Sébastien Rougier, Sabrina Guichard and Hugues Abriel corrected the manuscript. All approved the final version of the manuscript and agree to be accountable for all aspects of the work in ensuring that questions related to the accuracy or integrity of any part of the work are appropriately investigated and resolved. All persons designated as authors qualify for authorship, and all those who qualify for authorship are listed.

## CONFLICT OF INTEREST

None declared.

## Supporting information




**Supporting Information**: eph70294‐sup‐0001‐FigureS1.tif

**Supporting Information**: eph70294‐sup‐0002‐SuppMat.docx

## Data Availability

All raw Western blots are available upon request from the corresponding author, Dr. Rougier Jean‐Sébastien.
