# Supplementary material for: Glycosylation of the murine cardiac channel TRPM4 is altered by the pathogenic p.I376T variant
Source: Exp Physiol. 2026 Apr 29;111(7):3294–309. doi: 10.1113/EP093873 (PMC13327340; doi:10.1113/EP093873)
Supplement: Supplementary file 2 — Supporting Information: eph70294‐sup‐0002‐SuppMat.docx [file EPH-111-3294-s002.docx]

# SUPPLEMENTARY

# Glycosylation of the murine cardiac channel TRPM4 is altered by the pathogenic p.I376T variant

## Sabrina Guichard^1£^, Emanuele Di Lorenzo^1£^, Dominic Michiel Schneiter^1^, Maria Essers^1^, Prakash Arullampalam^1^, Jean-Sébastien Rougier^1*^, and Hugues Abriel^1*^

^1^ Institute of Biochemistry and Molecular Medicine, University of Bern, Bühlstrasse 28, 3012, Bern, Switzerland

* Correspondence should be addressed either to:

Rougier Jean-Sébastien, PhD or Abriel Hugues, MD, PhD

Institute of Biochemistry and Molecular Medicine, University of Bern

Bühlstrasse 28, CH-3012 Bern, Switzerland

E-Mail: jean-sebastien.rougier@unibe.ch (ORCID: 0000-0002-8710-0260)

E-Mail: hugues.abriel@unibe.ch (ORCID: 0000-0003-0465-5138)

# ^£^Both authors contributed equally to this work.

**SUPPLEMENTAL FIGURE LEGENDS**

**Supplementary Figure 1. *Trpm4* ^I376T/I376T^ C57BL6/JRj mouse model generation.** *A:* Diagram showing the strategy used to generate the new mouse model. A 9.1 kb genomic DNA used to construct the targeting vector was first subcloned from a positively identified C57BL/6 BAC clone. The region was designed such that the long homology arm (LA) extends ~5.65 kb 5’ to the 5’ Lox P cassette, and the short homology arm (SA) extends about 2.51 kb 3’ to the insertion of the inversion cassette. The inversion cassette is flanked by two mutant Lox sites (Lox 71/66) and consists of the mutant exon 9 (ATC → ACC) and the flanking genomic sequences from upstream of exon 7 to downstream of exon 9 for correct splicing (Inv sdEx7-9*Sa). This cassette was inserted in the reverse orientation within introns 9 and 10. The FRT-flanked Neo cassette was inserted immediately upstream of the inversion cassette, 148 bp from wild-type exon 9. The target region spans 914 bp and contains exons 7-9.
